# Zebrafish as an Alternative Vertebrate Model for Investigating Developmental Toxicity—The Triadimefon Example

**DOI:** 10.3390/ijms18040817

**Published:** 2017-04-12

**Authors:** Maria Zoupa, Kyriaki Machera

**Affiliations:** Laboratory of Toxicological Control of Pesticides, Department of Pesticides Control and Phytopharmacy, Benaki Phytopathological Institute, Attika 44561, Greece; m.zoupa@bpi.gr

**Keywords:** zebrafish, alternative model, developmental toxicity, triadimefon

## Abstract

Triadimefon is a widely used triazole fungicide known to cause severe developmental defects in several model organisms and in humans. The present study evaluated in detail the developmental effects seen in zebrafish embryos exposed to triadimefon, confirmed and expanded upon previous phenotypic findings and compared them to those observed in other traditional animal models. In order to do this, we exposed embryos to 2 and 4 µg/mL triadimefon and evaluated growth until 120 h post-fertilization (hpf) through gross morphology examination. Our analysis revealed significant developmental defects at the highest tested concentration including somite deformities, severe craniofacial defects, a cleft phenotype along the three primary neural divisions, a rigorously hypoplastic or even absent mandible and a hypoplastic morphology of the pharyngeal arches. Interestingly, massive pericardial edemas, abnormal shaped hearts, brachycardia and inhibited or absent blood circulation were also observed. Our results revealed that the presented zebrafish phenotypes are comparable to those seen in other organism models and those derived from human observations as a result of triadimefon exposure. We therefore demonstrated that zebrafish provide an excellent system for study of compounds with toxic significance and can be used as an alternative model for developmental toxicity studies to predict effects in mammals.

## 1. Introduction

Triazole compounds belong to a broad group of fungicides used widely for the control of infectious diseases of both plants and humans [1]. To date, about 40 different triazole agrochemicals are retailed worldwide, where more than 20 different compounds are produced for medical use [2]. The concern on the impact of triazole usage to the natural environment and in human health has been increasingly raised in the past years as mammalian studies reveal developmental disruptions, whereas combination of morphological and molecular data is essential to promote our knowledge on the mechanism of action [1,3]. In this work, triadimefon, a triazole commonly used as a food preservative, an anti-microbial agent and as a plant protection product, was chosen as the preferred compound for phenotypic comparison between traditional animal models and the zebrafish organism.

The developmental effects of triadimefon have been tested in various model organisms using both in vivo and in vitro approaches. Triadimefon exposure can affect mammalian species by causing multiple toxic effects on liver, thyroid, reproductive system as well as carcinogenicity and teratogenicity [4,5,6,7]. In rodents, triadimefon causes hepatocyte hypertrophy in a dose dependent manner [8] whereas in rats it has been found to act as a thyroid tumorigen [9]. In addition, triadimefon treated rats exhibited reduced fertility and decrease in size in two generation reproduction studies [10]. It has also be shown to elicit a neurotoxic syndrome in rats characterized by increased motor activity [11]. Strong evidence that triadimefon can disturb embryo development arise from several embryogenesis studies. The treatment of mouse embryos with triadimefon during the early embryogenetic period induced craniofacial malformations such as reduction and fusion of the first and second branchial arches [12,13,14] and axial deformities [12]. Interestingly, maxillary ectopic cartilage, similar to that observed after Retinoic acid (RA) exposure [15], has been reported after in vivo triadimefon treatment of mouse embryos [12]. Other studies have also suggested similar phenotypes in Xenopus [16]. Moreover, work with the ascidian *Phallusia mammillata* revealed malformations caused by exposure of embryos to triadimefon on early development in a dose dependent manner [17].

The zebrafish (Danio-rerio) is considered to be a promising model for predicting developmental toxicity in vertebrates including humans [18]. The zebrafish embryo is an alternative and fast model for screening chemicals that combines the benefits of an in vivo model with the advantages of an in vitro approach. Its developmental process that is highly comparable to that of mammals, its complete organ formation by 120 hpf and its transparency that allows easy visualization of phenotypic effects makes zebrafish an effective animal model for toxicological research [18,19].

In this study, we performed detailed phenotypical analysis in an initial attempt to evaluate developmental deformities arising to zebrafish embryos exposed to 2 and 4 μg/mL triadimefon from 24 to 120 hpf. The most pronounced deformities were seen in the embryos treated with 4 μg/mL triadimefon at 120 hpf. Those included somite formation irregularities, disorganized muscle fibers and hypoplastic horizontal myoseptum, hypomorphic midbrain, flattened forebrain, a severe cleft phenotype along the primary neural divisions and hypoplastic or even absent mandible. Interestingly, massive pericardial edema, abnormal shaped heart, brachycardia and inhibited or even absent embryonic blood circulation system were further identified. This study allowed us to compare our results with those observed in mammalian models, shed light on phenotypes arisen from triadimefon exposure and demonstrate the importance of zebrafish as a model for alternative testing in the field of developmental toxicology.

## 2. Results and Discussion

### 2.1. Observations on Hatching Rate and Length Growth

**Figure 1:**
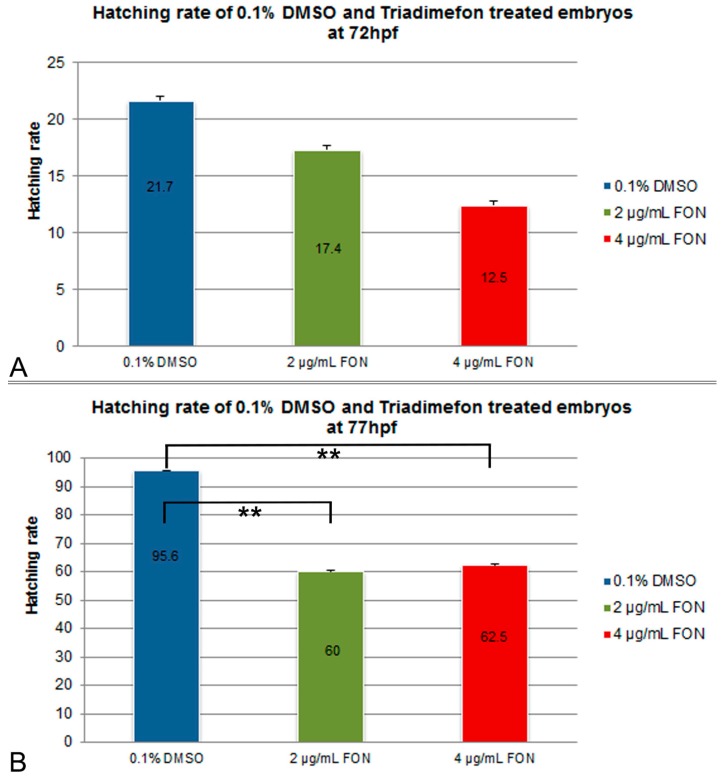
Effects of triadimefon exposure on zebrafish hatching process. Bar charts illustrate hatching rates of zebrafish embryos exposed to 0.1% DMSO, 2 and 4 μg/mL triadimefon concentrations (**A**) at 72 hpf and (**B**) 77 hpf. Triadimefon exposed embryos exhibit a concentration dependent decrease in hatching rate (17.4% and 12.5% in 2 and 4 μg/mL triadimefon treated embryos respectively) when compared to controls (21.7%) at 72 hpf. Asterisks in B denote statistical significance differences of 2 and 4 μg/mL triadimefon exposed groups relative to solvent controls at ** *p* < 0.001. The total number of embryos examined in the control, 2 and 4 μg/mL triadimefon concentration groups were 89, 62 and 67 respectively. FON, triadimefon. Error bars indicate standard deviations.

For the assessment of effect of the model substance triadimefon on the hatching rate, treated embryos were compared to controls exposed to 0.1% DMSO at 72 and 77 hpf. Hatching in the two concentrations of triadimefon began at 48 hpf and continued until 96 hpf when the hatching was complete. At 72 hpf, hatching rate of embryos exposed to 2 and 4 μg/mL triadimefon was 17.5% and 12.5% respectively, compared to 21.7% of the control (Figure 1A). At 77 hpf, hatching was statistically significantly lower, i.e., 60% and 62.5% respectively at the two tested concentrations (*p* = 0.004 and *p* = 0.006 respectively) when compared to the almost fully hatched control embryos (95.6%; Figure 1B). Therefore, the most significant retardation in the hatching rate of zebrafish embryos was observed at 77 hpf at both tested concentrations of 2 and 4 μg/mL of triadimefon.

**Figure 2:**
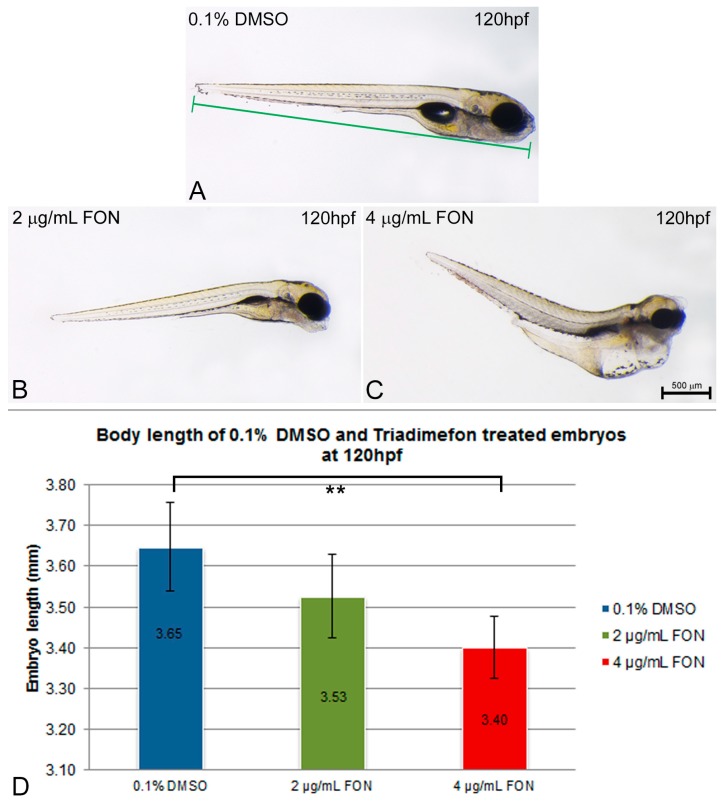
Zebrafish embryo length is affected by triadimefon exposure. (**A**–**C**) Examples of measurements of embryo length from 0.1% DMSO control (**A**), 2 and 4 μg/mL triadimefon (**B**,**C** respectively) at 120 hpf. Embryos shown to the same scale, indicated by the scale bar (=500 µm in **C**). (**D**) Bar chart illustrates average body length in 0.1% DMSO controls, 2 and 4 μg/mL triadimefon treated embryos, at 120 hpf. Body lengths of triadimefon treated embryos decreased in a dose-dependent manner. The highest triadimefon concentration exposure leads to a statistically significant length reduction of 4 μg/mL triadimefon exposed embryos as compared to the control group. The number of embryos examined in the control, 2 and 4 μg/mL triadimefon concentration groups were 45, 31 and 36 respectively. Asterisk(s) denote statistical significance; ** *p* ≤ 0.01. FON, triadimefon. Error bars indicate standard deviations.

Body length was evaluated as an indication of effect on growth at 120 hpf. The length was measured in control embryos and compared to the 2 and 4 μg/mL triadimefon treated embryo lengths (Figure 2A vs. Figure 2B,C). The average body length of zebrafish embryos at 2 μg/mL triadimefon was 3.53 mm compared to control (3.65 mm; Figure 2D). Exposure to 4 µg/mL triadimefon resulted in the shortest observed body length of 3.40 mm (statistically significant reduction *p* = 0.001, Figure 2D).

In conclusion, the exposure of zebrafish embryos to the model compound, known to produce perturbations in the embryonic development, resulted in statistically significant retardation of hatching from the chorion than those of the control 0.1% DMSO solution, as well as in growth retardation at the highest dose tested. The delayed hatching observed in experimental embryos could be due to differences in the embryo movement activity within the egg, since embryos exposed to triadimefon showed a decrease in the frequency of spontaneous movements within the chorion [20]. The temporary delay in hatching could also be associated with the decrease in body lengths seen in triadimefon treated embryos. Toxic effects on somitic development, skeletal and/or muscle development, all phenotypes discussed below in this paper, might be the causative reason behind the shortening of body length [21].

### 2.2. Skeletal and Soft Tissue Effects

**Figure 3:**
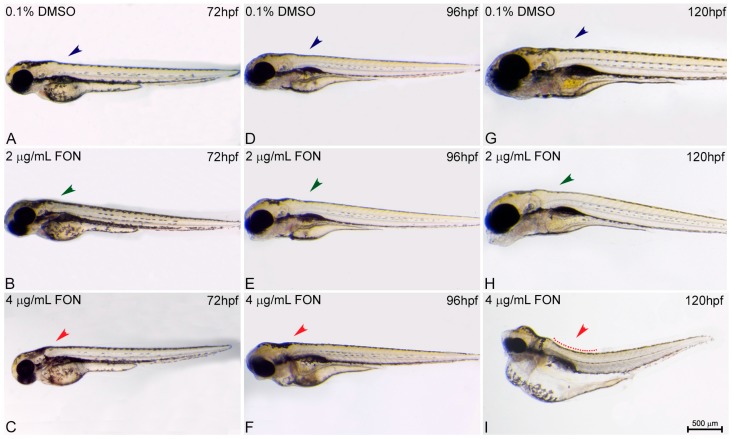
Triadimefon exposed embryos exhibit bend spine defects. Representative morphological evaluation and comparison of control (*n* = 45) and triadimefon treated embryos (31 and 36 embryos examined) (**A**–**C**) at 72 hpf, (**D**–**F**) 96 hpf and (**G**–**I**) 120 hpf. 72 hpf zebrafish embryos exposed to 2 and 4 μg/mL triadimefon show normal development of spine (left panel; green and red arrowheads in **B**,**C** vs. blue in **A**). A still comparable spine morphology is seen for all embryos at 96 hpf (middle panel; blue, green and red arrowheads in **D**–**F**). While, no malformations of neural spine were observed in 2 μg/mL triadimefon exposed embryos (green arrowhead in **H**), the spine of 4 μg/mL triadimefon embryos was severely curved (red arrowhead and dotted region in **I**, 34/36 embryos examined) at 120 hpf. FON, triadimefon. Embryos are shown to the same scale (bar = 500 µm in **I**).

For the study of zebrafish potential response and comparison to the effects already known from other animal models using triadimefon as a compound of choice the structure of embryo spine and potential presence of curvature was assessed at 72, 96 and 120 hpf. At 72 hpf, comparison of control and triadimefon exposed embryos at both tested concentrations, showed no changes in spine development (Figure 3A vs. Figure 3B,C; blue, green and red arrowheads respectively). One day later, the normal phenotypic pattern was still observed across all embryos (Figure 3D–F). In contrast, at 120 hpf, a severe curvature of the spine was observed at the highest triadimefon concentration of 4 μg/mL (Figure 3I red), while no spine defects were identified at 2 μg/mL triadimefon versus control embryos (Figure 3G–H). Thus, the concentration of 4 μg/mL triadimefon is a clear effect concentration as it relates to the induction of spine defects in zebrafish embryos at 120 hpf. This observation contrasts the Liu et al., remarks in which triadimefon induced bent spine defects were seen at a concentration as low as 2 μg/mL [20].

Developmental defects of neural structures such as the spine can be associated with early defects in somite formation that in turn leads to deformities of the muscle and skeleton [18]. A common endpoint for assessing zebrafish toxicity is the muscle fiber development and organization. For example, sodium benzoate exposure results in disruption of muscle fiber organization [22] whereas abnormalities in fiber length is a phenotypic result of ethanol exposure [23]. Axial skeleton also derives from somites and develops beyond 72 hpf. Again, skeletal deformities are observed after treatment with benomyl, a systemic benzimidazole fungicide [24]. Importantly, a study on CD-1 mice treated with triadimefon revealed alterations at the axial skeletal level of embryos that could be explained by abnormal somite specification [12], also observed in zebrafish (data discussed later in this study).

To date, there are not known mechanisms that account for the defective spine in triadimefon or triazole phenotype. The bent spine defects could be attributed to a reduction in myosin and myotome formation critical for a robust musculoskeletal system formation. In this work, a correlation between the zebrafish bend spine phenotype and the presence of skeletal abnormalities detected at the axial level (fused and cleaved or asymmetric vertebrae) of mice exposed to triadimefon could occur [12,13] and will be discussed later in this paper.

**Figure 4:**
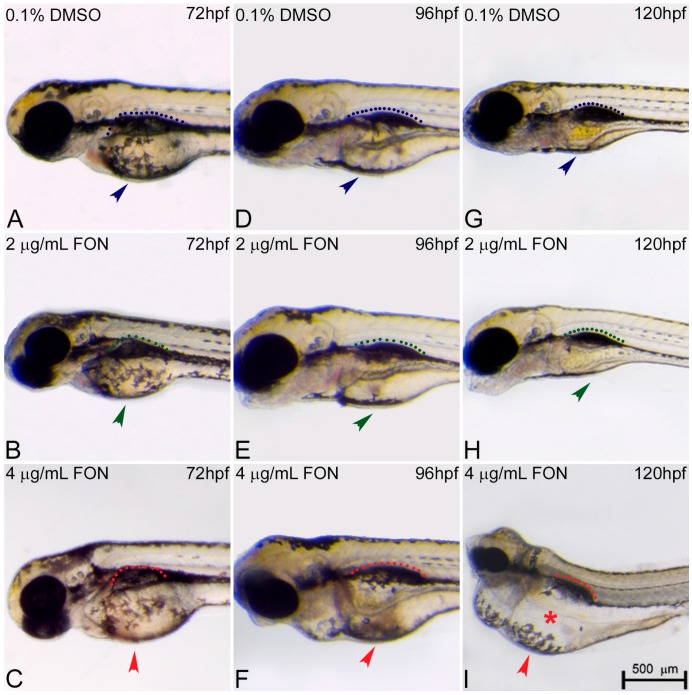
Triadimefon treated embryos are characterized by yolk sac edema and swim bladder abnormalities. Representative morphological evaluation of embryos (**A**–**C**) at 72 hpf, (**D**–**F**) 96 hpf and (**G**–**I**) 120 hpf. At 72 hpf, embryos exposed to 2 and 4 μg/mL triadimefon show normal development of yolk sac and swim bladder (left panel; compare green and red compared to blue arrowhead and dotted region). At 96 hpf (middle panel), 2 μg/mL triadimefon embryos exhibit minor yolk sac enlargement (green arrowhead in **E**, 77/80 embryos) and minor inhibition of swim bladder development (green dotted region in **E**, 74/80 embryos), while in 4 μg/mL triadimefon embryos, yolk sac and swim bladder development is perturbed (red arrowhead and dotted region in **F**, 89/89 embryos). At a progressed developmental stage (right panel), the 2 μg/mL triadimefon exposed embryos demonstrate minor yolk sac defects and an induced uninflated swim bladder (green arrowhead and dotted region in **H**, 77/80 and 74/80 embryos respectively). The most prominent abnormalities at 120 hpf are detected in the 4 μg/mL triadimefon treated embryos where the yolk sac is not depleted and the swim bladder is uninflated (red arrowhead, asterisk and dotted region in **I**, 89/89 embryos). FON, triadimefon. Embryos are shown to the same scale (bar = 500 µm in **I**).

The morphological changes observed in the developing embryo also included abnormalities in yolk sac and swim bladder development. At 72 hpf, triadimefon treated embryos did not present any developmental anomalies of the yolk sac and swim bladder compared to control embryos (Figure 4A–C; arrowhead for yolk sacs, dotted regions for swim bladders). One day later, 2 μg/mL triadimefon treated embryos presented minor yolk sac enlargement and minor swim bladder defects (Figure 4E green arrowhead and green dotted region compared to Figure 4D blue arrowhead and blue dotted region). A more enlarged yolk sac and minor inhibition of swim bladder was observed with increasing triadimefon concentration to 4 μg/mL, compared to 2 μg/mL embryos (Figure 4F vs. Figure 4E) and to control for animals exposed at the same hpf (Figure 4F vs. Figure 4D). When exposure took place at 120 hpf, minor yolk sac malformations and an induced uninflated swim bladder was identified at 2 μg/mL triadimefon treated embryos (Figure 4H; vs. Figure 4G). Macroscopically examined embryos exposed to 4 μg/mL triadimefon concentration revealed severe structural defects. The most evident were enlarged and misshaped yolk sac and uninflated swim bladder (100% *n* = 89 embryos), in comparison to the controls (100% *n* = 92; Figure 4I vs. Figure 4G). This suggests that increases in malformations severity are coupled to increasing triadimefon concentration. Morphological analyses of triadimefon embryos during the course of three developmental stages studied here revealed non-depleted yolk sac and uninflated swim bladder, two phenotypes that arose at 96 hpf and were clearly identified by 120 hpf.

At the first 96 h of the zebrafish development, the yolk sac plays an important role as the main source of nutrients by ensuring embryo proper growth and survival. During this process, lipids enter the embryo at the yolk and embryo interface region named yolk syncytial layer. This layer transports lipids from the yolk to the embryo and once the circulatory system is fully functional they are transported to target tissues via the bloodstream [25]. The yolk sac resorption is evident approximately at 120 hpf, with total depletion at around 168 hpf, a time point where the larvae must acquire lipids via food [26,27]. Yolk sac abnormalities may therefore affect lipid metabolism deficiency leading to developmental delay of treated embryos. Several different animal studies for developmental toxicity have shown the association of triazole compounds and lipid homeostasis. A toxicogenomic analysis of rat liver and human primary hepatocytes revealed that myclobutanil, propiconazole and triadimefon affect fatty acid catabolism, bile acid and triazole metabolite transport [3]. A transcriptomic study on the effect of different flusilazole exposures on the zebrafish embryonic genome revealed gene expression responses of fatty acid metabolism [28]. Finally, Hermsen et al., analyzed gene expression of five 1,2,4-triazole derivatives with different potencies, the cyproconazole, hexaconazole, triadimefon, myclobutanil and triticonazole. In the same work, triadimefon exposed embryos were found to be significantly enriched for nuclear receptors in lipid metabolism and toxicity pathway genes [29]. To summarize, an association between the yolk sac defects revealed after triadimefon exposure using our zebrafish experimental model and lipid metabolism defects seen in the aforementioned studies could be plausible.

In addition to lipid metabolism, yolk development has been associated with endocrine disrupting chemicals and yolk sac phenotype is one of the 120 hpf specific endpoints affected by thyroid-active compounds [18]. Thyroid hormones play important roles in regulation of development and growth, energy provision, metabolism and reproduction [30]. Zebrafish thyroid hormonal homeostasis studies revealed a linkage between triadimefon exposure and altered gene expression of the hypothalamic-pituitary-thyroid axis leading to a disruptive mechanism of synthesis, regulation, and action of thyroid hormones [31]. This indicated that triazole fungicides might have the abilities to act as endocrine disruptors via a range of mechanisms. Yet a clear correlation between the observed yolk phenotype and thyroid disruption in triadimefon treated embryos remains to be elucidated.

To date, a morphological and transcriptional homology between the mammalian lung and amphibian swim bladder has been well established [32]. To our knowledge, mammalian defects in lung morphogenesis and development after triadimefon exposure have not been reported yet making a direct comparison between zebrafish and other animal models not feasible at this stage.

The zebrafish swim bladder arises early in larval development as an invagination of the foregut and initially forms as a single chamber. After formation, and in order to inflate their swim bladder, zebrafish larvae must have access to an air-water interface where they gulp air. During zebrafish adulthood, the bladder is a gas- filled sac able to regulate its gas volume to adjust body density and buoyancy after changing depth in the water column. This competence allows a significant reduction in metabolic cost since the zebrafish does not need to expend energy by swimming to maintain its normal vertical position [33]. In the present work, a mild effect on swim bladder phenotype initiated at 96 hpf in both triadimefon exposed embryo concentrations. By 120 hpf, the uninflated swim bladder was induced at the low concentration of 2 μg/mL triadimefon treated embryos, with the most severe effect on phenotype seen at 4 μg/mL. A hypothesis could be that the abovementioned swim bladder could at least partially be associated with the inability of triadimefon treated embryos to reach promptly full hatching stage, subsequently not allowing them to proceed with inflation. A previous study has measured the locomotion activity in embryos and larvae under the influence of 2 and 4 μg/mL triadimefon [20]. According to the authors, embryos exposed to 4 μg/mL triadimefon displayed reduced movement at 24 hpf. A decrease in swimming speed in a dose dependent manner at later developmental stages was also observed. This observation is in line with the hypothesis that inhibited embryo movement with a curved spine and a muscle/skeletal alteration interferes with the embryos’ ability to swim towards the surface and access the air-water interface, leading to a severe swim bladder deficiency.

### 2.3. Alternations in Somitic, Myotome and Horizontal Myoseptum Formation Following Triadimefon Exposure

**Figure 5:**
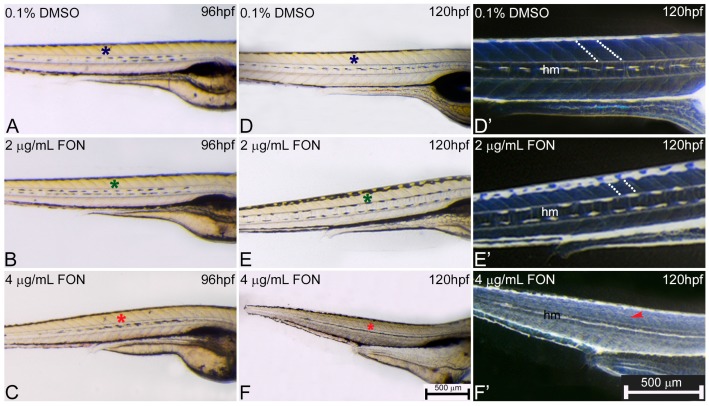
Abnormal somitic, myotome and horizontal myoseptum phenotypes are described in triadimefon exposed zebrafish embryos. Representative morphological evaluation of zebrafish control (*n* = 92) and triadimefon treated embryos (80 and 89 embryos examined respectively) (**A**–**C**) at 96 hpf, and **(D**–**F**, **D’**–**F’**) from 96 to 120 hpf. At triadimefon concentrations of 2 and 4 μg/mL somitic and myotome development appear to be normal (left panel; green and red asterisks in B and C). One day later (middle panel), gross morphology evaluation shows no alterations of the myotome and somitic structures in 2 μg/mL triadimefon treated embryos (green asterisk in **E**). However, 4 μg/mL triadimefon embryos at 120 hpf present severe altered somitic structures with no clear boundaries (89/89) and disorganized muscle fibers (red asterisk in **F**) (85/89). Inversion of **D**–**F** embryo figures (left panel; **D’**–**F’**) reveals the absence of clear somitic boundaries (red arrowhead in **F’**) compared to 2 μg/mL triadimefon—treated and control embryos (white dotted region in **E’**). The horizontal myoseptum (hm) is normally developed in 0.1% DMSO control and 2 μg/mL triadimefon—treated embryos (**D’**,**E’**) but severely hypoplastic in embryos exposed to 4 μg/mL triadimefon (**F’**). FON, triadimefon. Embryos in **A**–**F** are shown to the same scale (bar = 500 µm in **F**) while embryos in **D’**–**F’** are shown to the same scale (bar = 500 µm in **F’**).

For further investigation of potential correlation between zebrafish and other classical animal models after triadimefon exposure and the observed effects on the muscular phenotype, the presence and morphological development of somites, myotome and horizontal myoseptum at 72, 96 and 120 hpf were evaluated. At 72 hpf, control and triadimefon exposed embryo comparison did not reveal any somitic abnormalities [34]. At 96 hpf, myotome and somitic development in controls was comparable to 2 and 4 μg/mL triadimefon embryos (Figure 5A–C; asterisks) displaying a normal phenotypic pattern under gross morphology examination. Further evaluation at 120 hpf, revealed no developmental alterations of somites (Figure 5E green asterisk) and myotome (Figure 5E’ white dotted lines) at 2 μg/mL when compared to control embryos (Figure 5D,D’ respectively). At the same stage and at 4 μg/mL triadimefon, embryos exhibited severely altered somitic structures with no clear boundaries and disorganized muscle fibers compared to controls (Figure 5F,F’ vs. Figure 5D,D’; Videos S1 and S2 vs. Video S3). Finally, the horizontal myoseptum, the connective tissue partition developing at the apex of the chevron-shaped myotome, was drastically reduced in embryos exposed to 4 μg/mL triadimefon (Figure 5F’) compared to embryos exposed to 2 μg/mL triadimefon (Figure 5E’) and controls (Figure 5D’). To the best of our knowledge this is the first description of triadimefon induced irregularities in somite formation, the disorganized muscle fibers and hypoplastic horizontal myoseptum in zebrafish at 4 μg/mL.

In zebrafish, somitic formation initiates with the segmentation of paraxial mesoderm and continues with the formation of morphologically distinct somites via the epithelization of presogmitic segments. The myotome is a group of muscles that contain fibers and gives rise to zebrafish musculature, whereas the horizontal myoseptum is a fibrous sheet that divides the myotome into a dorsal and a ventral part firstly detectable at around 28 hpf [35,36]. Zebrafish are greatly supported by the buoyancy of water and swim bladder and do not require an extensive skeleton. To compensate for the lack of a robust skeleton, they require a large muscular system that allows mobility through a relatively viscus aquatic environment. As a result, proper muscle system formation and function is critical to zebrafish life [33].

Studies performed in *X. laevis*, have previously uncovered a connection between triadimefon exposure and teratogenic effects in craniofacial structures. Embryos exposed to triadimefon at the neural stage presented irregular muscle pattern associated with the 1st and 2nd branchial arches [16]. Another study on *X. laevis* also revealed reduction of the net like structures at the level of hypaxial muscles as a result of triadimefon exposure. The severity of phenotypic disruption was strongly associated with the concentration levels utilized during exposure [37]. The observed muscle phenotype may possibly be a result of a triadimefon toxic event, a direct effect of altered signaling or a secondary effect due to the presence of a severe embryonic edema. It is interesting to note that triadimefon exposure has been shown to affect and disrupt the RA pathway resulting to craniofacial abnormalities [38]. It is likely that in the case of the present study, triadimefon exposure may also affect RA content during embryo morphogenesis leading to a muscle phenotype.

To date, a zebrafish mutant that displays horizontal myoseptum defects has been identified [39]. In this mutants, the basic segmentation of the paraxial mesoderm proceeded appropriately, but the differentiation of slow muscle cells was prevented leading to flat or U-shaped somites [39,40]. It would therefore be interesting to investigate in detail the differentiation of slow muscle cells in triadimefon treated embryos in an attempt to identify the mechanism underling the somite phenotype. Moreover, potential differences between the genetic control of somitogenesis could be identified as recent publications have shown a role of adaxially expressed integrins in affecting somite boundary formation and maintenance through different molecular mechanisms [41,42]. Eeden et al., have identified genes that affected somitic formation and patterning in zebrafish. Mutants in six genes *you*, *you-too*, *chameleon*, *sonic-you*, *u-boot* and *choker* had absent or a reduced horizontal myoseptum and U-shaped somites [40]. The expression patterns of the abovementioned genes could be potential targets of the triadimefon altered gene pathway during myoseptum formation. Additionally, if the muscle pioneers are required for myoseptum formation, mutants that lack that structure might also have absence the muscle pioneers. Thus, it would be valuable to study potential abnormalities of muscle pioneers in the triadimefon exposed embryos.

Disorganized muscle fibers and abnormalities in somitic development detected in the present study may explain in part the previously reported abnormal spontaneous movement of triadimefon treated embryos [20]. In addition, irregularities in somitic boundaries along with the disorganized muscle fibers could be the primary cause contributing to the body length reduction of triadimefon exposed embryos. Developmental defects of neural structures, such as the triadimefon spine curvature phenotype, could also be attributed to early defects in somatic formation leading to deformities of the muscle and skeleton. In summary, the muscle/somitic phenotype, after zebrafish exposure to triadimefon, revealed an altered muscle development in the present study and it is in line with the effects observed on *X. laevis* studies [16,37], suggesting that zebrafish is a valuable alternative model for screening muscle defects.

### 2.4. Triadimefon Leads to Craniofacial and Pharyngeal Arch Defects in Zebrafish Embryos

Another endpoint of the present work was the evaluation of the craniofacial and pharyngeal arch defects after zebrafish embryo exposure to triadimefon and comparison of those effects to effects observed on the currently used animal models. Initially, and in order to test the potential effects of different triadimefon concentrations on zebrafish craniofacial development and pharyngeal arch morphology, embryos were analyzed for deformities through gross morphology evaluation during the third, fourth and fifth day of development.

**Figure 6:**
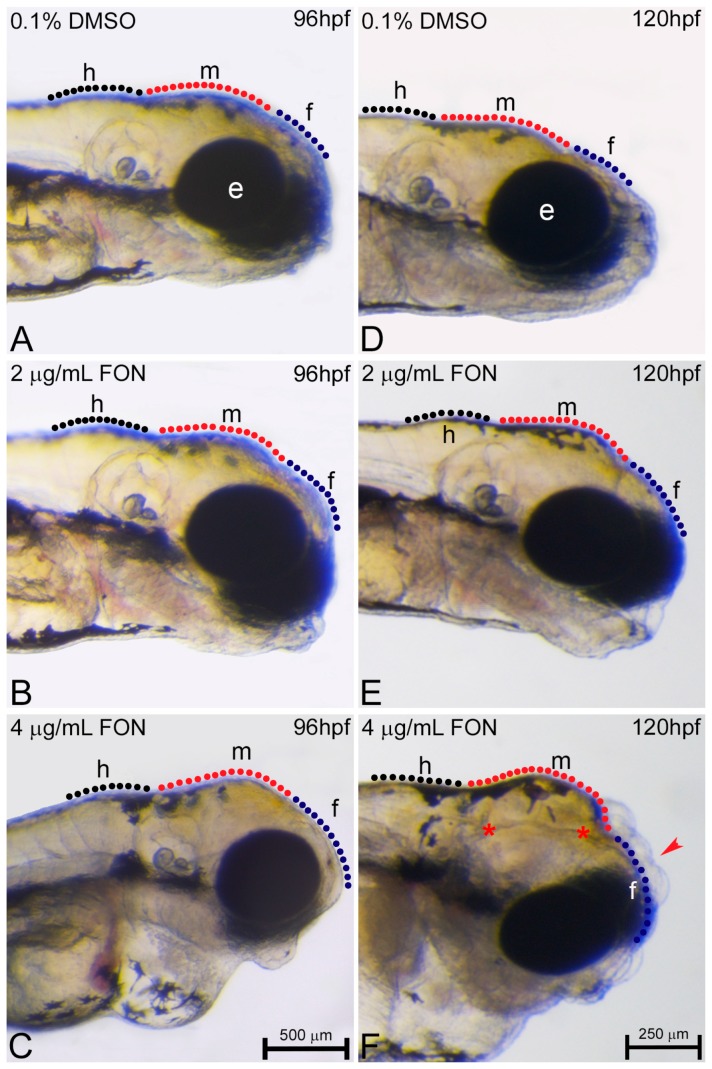
Triadimefon exposed embryos display cranial defects. Representative morphological evaluation of embryos (**A**–**C**) at 96 hpf and (**D**–**F**) 120 hpf. 2 μg/mL triadimefon treated embryos present normal brain development at 96 hpf (**B**). At 4 μg/mL the first sings of cranial forebrain-midbrain morphological alterations are observed (**C**, 89/89 embryos). Morphological alterations at 2 μg/mL triadimefon treated embryos at 120 hpf include a decrease of the average brain size with the forebrain developing a compacted-like form (66/80 embryos), whereas midbrain development is slightly delayed (64/80) (**E**). A more pronounced phenotype is observed at the same stage with 4 μg/mL triadimefon (**F**). Deformities were of high severity and comprised of severely hypoplastic forebrain (red arrowhead in **F**, 80/89), decreased midbrain size lacking forebrain-midbrain boundary (89/89) and delay of hindbrain development (89/89). A high proportion of embryos also exhibited severe cleft of the anterior nervous system (red asterisks, 74/89). Dotted blue, red and black lines in **A**–**F** map the forebrain, midbrain and hindbrain cranial structures respectively. FON, triadimefon. Embryos in **A**–**C** are shown to the same scale (bar = 500 µm in **C**), while embryos in **D**–**F** are shown to scale (bar = 250 µm in **F**).

No abnormalities were detected at 72 hpf in the craniofacial morphology of triadimefon treated embryos [34]. Cranial specific abnormalities were initially observed in embryos treated with 4 μg/mL triadimefon and consisted of a loss of forebrain-midbrain conformation with a flattened forebrain (Figure 6C vs. Figure 6A; blue and red dotted regions) at 96 hpf. To further investigate the triadimefon derived neurocranium defects, embryos were assessed at 120 hpf. In 2 μg/mL triadimefon, all three brain structures, forebrain, midbrain and hindbrain, were examined. The forebrain presented a more flatted-like shape, the midbrain was mildly affected by a developmental delay, whereas the hindbrain seemed to be the least affected cranial structure compared to controls (Figure 6E vs. Figure 6D). Embryos exposed to 2 μg/mL triadimefon exhibited a range of phenotypic effects with the one presented here being the most severe (82.9% of embryos phenotypically evaluated). On the other hand, 4 μg/mL triadimefon exposure revealed clear teratogenic effects that included a more pronounced cranial phenotype, when compared to controls as well as to 2 μg/mL triadimefon treated embryos (Figure 6F vs. Figure 6D,E). These malformations appear to be dose related and include severely reduced size and flattened forebrain, hypoplastic midbrain with undistinguishable forebrain-midbrain boundary and a minor interruption of hindbrain development. In addition, morphological evaluation revealed the presence of cleft forebrain-midbrain-hindbrain structures in 82.9% of 4 μg/mL triadimefon exposed embryos (Figure 6F; red asterisks).

**Figure 7:**
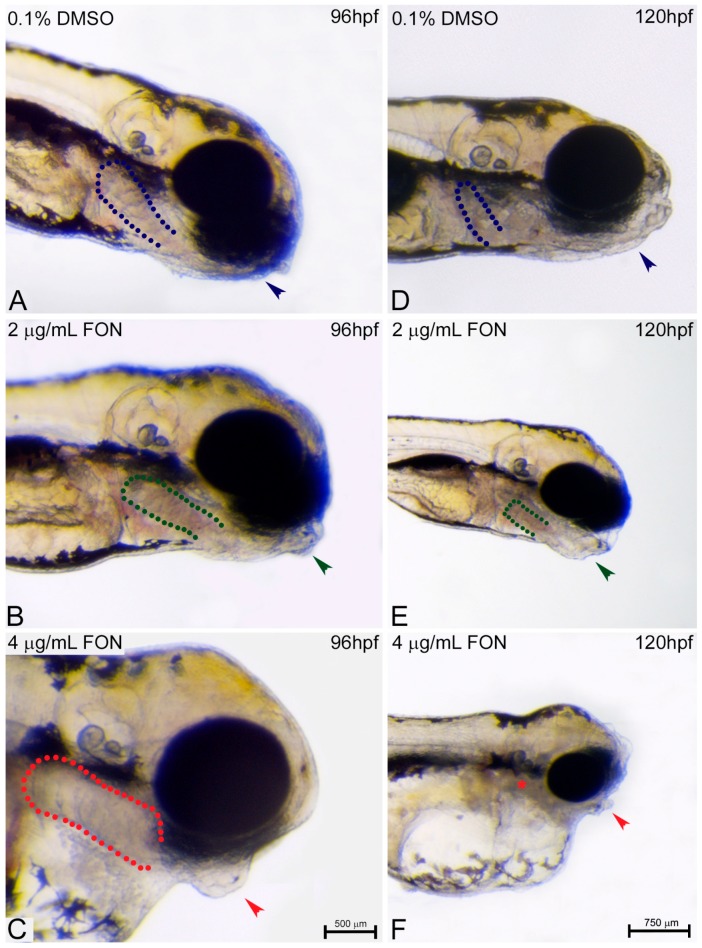
Pharyngeal arch development is affected by triadimefon. Representative morphological evaluation of the pharyngeal arches in embryos (**A**–**C**) at 96 hpf and (**D**–**F**) 120 hpf. Embryos at 2 μg/mL triadimefon (**B**) presented a minor protrusion that resulted from a developmental delay (green arrowhead, 73/80 embryos), whereas the pharyngeal arches remain unaltered (green dotted region 80/80). A more pronounced phenotype with a severely reduced mandible (red arrowhead in **C**, 84/89), but a still intact pharyngeal arch development (red dotted region, 89/89) was observed in 4 μg/mL triadimefon embryos (**C**). The pharyngeal arch development was still presented as intact (red dotted region in **C**, 89/89). 120 hpf zebrafish embryos treated with 2 μg/mL triadimefon (**E**) revealed hypoplastic mandible (73/80) and reduced size of pharyngeal arches (62/80) (green arrowhead and dotted region in **E**). The pharyngeal arch apparatus was severely reduced at the highest triadimefon concentration evaluated (red asterisk in **F**, 80/89), whereas mandibular processes were almost absent in all embryos examined (89/89) at 120 hpf. Triadimefon. Embryos in **A**–**C** are shown to the same scale (bar = 500 µm in **C**), while embryos in **D**–**F** are shown to scale (bar = 750 µm).

At 72 hpf evaluation of pharyngeal arch morphology of triadimefon treated embryos also revealed normal development [34]. A day later and at a triadimefon concentration of 2 μg/mL, pharyngeal arch structures presented a normal developmental pattern (Figure 7B; green dotted lines), whereas the mandibular and maxillary arches were delayed in development (Figure 7B; green arrowhead) compared to control (Figure 7A). Facial analysis at 4 μg/mL triadimefon, revealed a more pronounced mandibular arch phenotype and possible pharyngeal arch deformities, a phenotype that requires additional evaluation (Figure 7C vs. Figure 7A,B; arrowheads and dotted regions). Severe mandibular abnormalities were also observed, as expected, in 2 μg/mL triadimefon treated embryos at 120 hpf. The main malformation was the reduction of mandibular arch, whereas the pharyngeal arches were present and slightly hypoplastic (Figure 7E vs. Figure 7D; arrowhead and dotted lines respectively). Mandibular arch evaluation also revealed severely morphological alterations of the pharyngeal arch apparatus that included an almost complete absence of mandible and a firstly identified absence of pharyngeal arches, other than pharyngeal arch I, compared to control embryos (Figure 7C vs. Figure 7A; arrowhead and asterisk/dotted region). Concluding, the most pronounced cranial and pharyngeal deformities, in the present work, appear to be dose-dependent and include cranial and pharyngeal arch defects.

Craniofacial and skeletal defects from overexposure of triazole derivatives have been tested in traditional animal models using both in vivo and in vitro methodologies [43]. CD-1 female mice treated with 300 mg/kg of triadimefon carried fetuses with severe craniofacial and axial skeletal malformations. The defective structures observed were all derived from the first and second branchial arches of the pharyngeal apparatus and included defects of the middle ear ossicle, timpanic ring, squamosal and zygomatic bones, abnormal shape of bone and cartilaginous elements of the mandible as well as axial skeletal defects and in some cases cleft of the maxillary process [12]. Furthermore, CD-1 mice exposed to the highest levels of 500 mg/kg triadimefon on different gestation stages resulted in fetuses with axial skeletal malformations and cleft of the maxillae [13,44]. These defects were described as a result of the alterations in somitic organization and altered neural crest cell (NCC) migration from the encephalon to the frontonasal and anterior branchial arches. The axial abnormalities, on the other hand, could be explained due to the abnormal segmental identity specification in the hindbrain region [12,44]. Apart from animal model studies, human population case approaches revealed a possible correlation between high maternal methonidazole, an antibacterial antibiotic, administration and the presence of cleft palate in a small percentage of human fetuses [45,46]. Another triazole derivative, the fungicide cyproconazole has been shown to be a potent teratogen able to induce hydrocephaly, uretero-hydronephrosis and cleft of the maxillary arch in rat embryos after administration of 20 mg/kg/day [47,48].

In addition to the in vivo studies, the post implantation whole embryo culture (WEC) system from mouse or rat embryos has been utilized in an attempt to elucidate the triadimefon teratogenic potential during organogenesis. Rat WEC, at various triadimefon concentrations, resulted in the induction of specific concentration related effects of the first and second pharyngeal apparatus [49]. Another in vitro comparative study on the effect of triazole, flusilazole (an agricultural triazole fungicide) and fluconazole (a bis-triazole derivative) compounds on rat embryo cultures revealed reduction of the first branchial arch and agenesis of the second one [50]. A study of the teratogenic potential of the imidazole-derivatives ketoconazole and enilconazole carried out in rat WEC demonstrated a noticeable dysmorphogenetic activity on branchial arch apparatus that included hypoplasia, abnormally shaped or agenesis of the branchial arches [51]. TGF-β is another important regulator during early embryonic development and mediates a wide range of biological activities [52]. Disruption of TGF-β genes results in a variety of developmental defects, including craniofacial and cardiac defects [53]. Di Renzo and colleagues’ revealed that induction of teratogenic effects of triadiemfon results in TGF-β and CRABPI alterations in hindbrain rat culture explants [54]. We could therefore hypothesize that triadimefon exposed zebrafish embryos could present altered TGF-β pathway genes.

The action of different triazoles and its derivatives has also been studied in *X. laevis* embryos, which presented teratogenic effects under the influence of various triadimefon and triadimenol concentrations during neurulation phase. The aforementioned triazoles affected the *Xenopus* branchial apparatus at the level of muscles and cartilages of the maxillae and mandibular arches [16]. Comparing the anomalies caused by triazoles and by RA, it was concluded that similar to what has been shown for mammals [55,56,57], these observed malformations could be a result of excess endogenous RA content [16]. Papis et al., investigated the developmental window of sensitivity under triadimefon exposure in *Xenopus*. For this purpose, they treated larvae for a 2-h period at early gastrulation and neurulation and found the latter stage to be more sensitive to triadimefon exposure. Interestingly, all affected embryo analysis revealed alterations of the branchial arch derived cartilages, seen as hypoplasias, abnormal shape and agenesis of the branchial arches [58]. These phenotypes were in line with results detected in other animal model studies and further supported the hypothesis that triadimefon can interfere with NCC migration into the branchial mesenchyme leading to craniofacial abnormalities [58] mainly through Hox pathway alterations [38].

Finally, work on ascidian embryos exposed to triadimefon and imazalil has shown alteration of the central nervous system and papillary nerves [17]. The latter sensory vesicle inhibition could be compared to defective hindbrain seen in mammals after triazole exposure.

The zebrafish data from the present study are in line with the effects obtained from mouse, rat, *Xenopus*, ascidian Phallusia mammillata and in vitro studies; supportive of the conclusion that triadimefon is a potent teratogen able to induce craniofacial malformations and deformities of the pharyngeal apparatus. In addition, these results are suggestive of a common triadimefon mechanism of action that may involve strongly conserved molecules. Thus, it is demonstrated that the used experimental model provides an excellent alternative to mammalian system for the study and identification of potential developmental toxicants. In addition, the choice of zebrafish as model organism will furthermore permit the extension of the toxicological and teratogenic observations to explore novel pathways during the development of craniofacial complex and pharyngeal arches.

### 2.5. Defects in Cardiovascular Function of Triadimefon Exposed Embryos

Zebrafish embryos give also the possibility to further identify effects on cardiac morphology and function. In the present study, potential effects produced due to triadimefon exposure were compared to effects on the cardiac morphology and function observed from experiments from other animal model systems. The zebrafish heart and vasculature are two systems commonly studied in toxicity studies and usually manifesting as alteration in cardiac rhythm and reduction in blood flow thought-out the embryo. Impaired cardiovascular function as a result of triadimefon toxic effects was assessed in this work at three developmental stages, from 72 to 120 hpf, via gross morphology evaluation and heart rate quantification.

**Figure 8:**
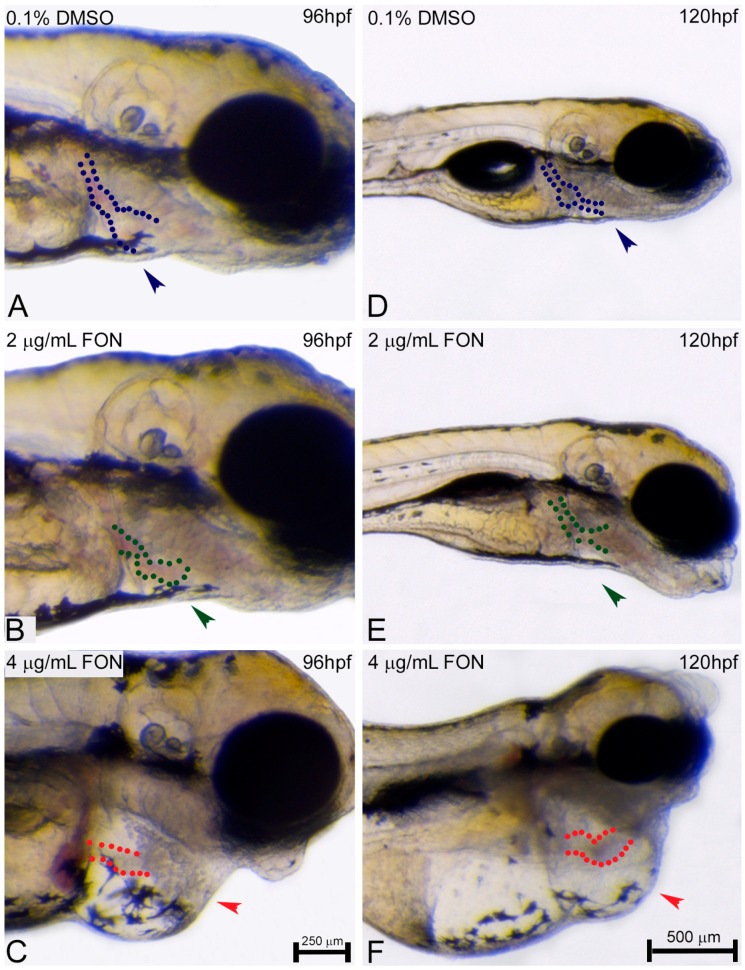
Cardiovascular defects induced by triadimefon. Representative morphological evaluation of the heart development in embryos (**A**–**C**) at 96 hpf and (**D**–**F**) 120 hpf. Embryos treated with 2 μg/mL triadimefon presented normal heart development at 96 hpf (green arrowhead in B indicated absence of edema (80/80); green dotted region represents the heart shape in **B**). Similarly, at 120 hpf heart morphological examination of this group showed normal heart development (**E**, 89/89). In contrast, at 4 μg/mL triadimefon (**C**, 80/89) cardiac defects manifested as heart edema (89/89) and a developmental delay of heart tube shape formation (80/89) (red arrowhead and dotted region respectively). A more pronounced phenotype consisting of substantial pericardial edema (red arrowhead, 89/89) and a linear heart tube (89/89) indicating heart malfunction (red dotted region) was the case for 120 hpf 4 μg/mL exposed embryos (**F**). Arrowhead indicates edema. Dotted blue, green and red lines in **A**–**F** map heart shapes. FON, triadimefon. Embryos in **A**–**C** are shown to the same scale (bar = 250 µm in **C**), while embryos in **D**–**F** are shown to scale indicated (=500 µm in **F**).

**Figure 9:**
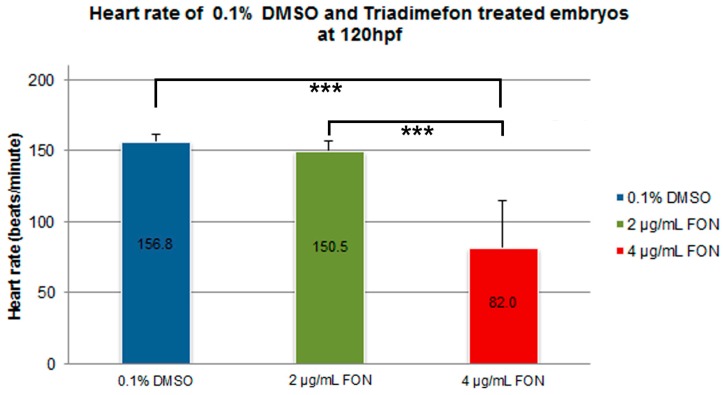
Heart rate in triadimefon treated embryos at 120 hpf. Heart rates in beats per minute (bpm) were counted in control (*n* = 20) and experimental embryos (18 and 16 embryos examined from the 2 and 4μg/mL triadimefon groups respectively). Embryos exposed to 2 μg/mL triadimefon presented a minor inhibition in heart rate (150.5 bpm). On the other hand, heart rate was drastically inhibited in 4 μg/mL triadimefon treated embryos (82.0 bpm), compared to 0.1% DMSO controls (156.8 bpm). Asterisks denote significant difference between triadimefon treatments and controls (*** *p* < 0.001). FON, triadimefon. Error bars indicate standard deviations.

Morphological evaluation at 72 hpf, revealed no adverse effects of triadimefon exposure on heart development and blood flow [34]. At 96 hpf, a normal phenotypic pattern of heart development was evident in 2 μg/mL embryos (Figure 8B vs. 8A; dotted region and arrowhead indicating absence of heart edema). Further evaluation at 120 hpf, did not disclose any developmental alterations such as heart shape or sac edema compared to control (Figure 8E vs. Figure 8D; dotted region and arrowhead). The first cardiac abnormalities, particularly pericardial edema (Figure 8C; red arrowhead) and a more linear heart tube (Figure 8C; red dotted region), arose in embryos at 96 hpf, exposed to 4 μg/mL triadimefon. At 120 hpf, these embryos exhibited severe cardiotoxicity with massive pericardial edema and a decrease in overall size of the heart development compared to control (Figure 8F vs. Figure 8D; arrowhead and dotted region, respectively). Additional evaluation of cardiac function encompassed quantification of triadimefon embryos’ heart rates at 120 hpf. In 2 μg/mL triadimefon treated embryos the number of heart beats was slightly reduced compared to the control group (Figure 9; Videos S4 and S5 respectively). The most prominent brachycardia was observed in embryos exposed to 4 μg/mL triadimefon where heat rate was decreased by 74.8 beats/min when compared to control embryo heart beats (Figure 9; Videos S5 and S6).

At the 120 hpf stage, alongside with cardiac function, blood circulation was also examined. Group comparison revealed severe circulation disruption in 5% (1/16) and complete circulation failure at 95% (15/16) of 4 μg/mL triadimefon treated embryos (Videos S1 and S2), compared to control (Video S3, *n* = 20). These observations show that the cardiac system and blood circulation were perturbed in triadimefon exposed zebrafish embryos and indicate that the development of cardiovascular system is another target of triadimefon. Table 1 summarizes the embryonic phenotypes observed after triadimefon exposure at 120 hpf.

In the present work, it is revealed that effects on cardiac and circulation function systems can also be detected on zebrafish model, as indicated from the triadimefon exposure at 4 μg/mL as early as 96 hpf. Triadimefon exposure resulted in the presence of severe pericardial edema, hypolastic/ abnormally shaped hearts, impaired heart rate and inhibited or even absent blood circulation.

Triazoles and their derivatives are compounds adversely affecting the cardiovascular system function. Carter et al., in a birth defect prevention study reported a possible elevated risk of hypoplastic left heart syndrome following exposure to first-trimester antifungal drugs miconazole, terconazole and ketoconazole [59]. Another work from Pulsen et al., reported a case in which a pregnant woman received fluconazole until the 7th week of gestation [60]. In this case, the infant had, amongst other defects, a congenital heart defect the so called, tetralogy of Fallot [60]. Although a number of studies on human subjects suggest a likely relationship between triazole exposure and malformations of infant hearts, these studies were not considered robust enough to establish a strong relationship. Nonetheless, several publications report cardiovascular abnormalities in animal models after exposure to different triazole compounds. In a reproductive toxicology screening of different compounds originally developed for therapeutic indications, two tested triazole compounds, JNJ1 and JNJ4, were able to induce pericardial edema in zebrafish embryos [61].

The first solid evidence that triadimefon exposure could contribute to cardiovascular defects came from mouse studies in which pregnant animals were administrated 500mg/kg triadimefon. Visceral examination of fetuses revealed the presence of great vessel abnormalities, interventriculal septal anomalies and dextrocardia, in a stage dependent manner [14]. The cardiovascular defects seen as a result to triadimefon exposure were related to disruption of cardiac NCC migration that leads in the outflow septation. Exposure to another triazole has also been shown to be phenotypically correlated with heart malformations. The clinically used antimycotic fluconazole has been shown to induce specific developmental anomalies, mainly at the level of branchial apparatus including induction of heart malrotation in whole rat embryos at concentrations of 125–250 and 500 µM after 48 h of in vitro exposure [50].

Currently, the pathogenic pathway postulated as the major cause of triazole craniofacial defects involves cytochrome P450 enzyme inhibition [54]. Several enzymes of P450 family are present during early morphogenetic events and alterations in their expression are associated with cardiovascular anomalies suggesting a developmental significance during formation of the heart and vasculature [62]. The specific inhibition of CYP26 isoenzymes is believed to alter endogenous RA concentration or its distribution, resulting in an altered RA cascade. Activation of an irregular RA signaling pathway triggers altered gene expression pattern of downstream targets during abnormal developmental stages. Several Tgfβ family genes are known to be inhibited during triazole exposure at multiple embryonic sites [54]. Tgfβ molecules are also known to mediate important biological activities including gene regulation of Hox family and are also involved in the specification and fate of NCC. Interestingly, targeted disruption of *Tgfβ* genes in different animal models resulted in the presence of cardiac malformations and affected a wide range of NCC- derived tissues [54]. Taking into account the above observations, we could hypothesize that the Tgfβ and Hox signaling pathways as well as P450 family enzymes could be altered during cardiovascular development under the influence of triadimefon in zebrafish embryos.

In the present work findings on zebrafish cardiovascular system (cardiac development, heart rate and blood circulation development) following triadimefon exposure are clearly demonstrated. Comparison of the results from the present study to those from the literature confirmed that triazole exposure alters cardiac development and function not only in traditional mammalian models such as the rat and in in vitro culturing approaches, but also in the zebrafish, making the latter a valuable alternative toxicological tool. Table 2 summarizes the phenotypes obtained from the zebrafish after triadimefon exposure and compares them to other triazole (including triadimefon) effects seen in other in vivo and in vitro animal models as well as in human studies.

## 3. Materials and Methods

### 3.1. Ethics Statement

All procedures were conducted in accordance to the revised directive 2010/63/EU (2010) on the protection of animals used for scientific purposes and according to zebrafish guidelines of the European Zebrafish Resource Centre (Karlsruhe Institute of Technology, Karlsruhe, Germany). The instructions are based on the principle of the Three Rs’, to replace, reduce and refine the use of animals used for scientific purposes (FELASA).

### 3.2. Fish Husbandry and Embryo Collection

Adult wild type fish (AB line) were maintained in a recirculating system (Zebtec benchtop, Techniplast, Buguggiate, Italy) in which the water was maintained at 28 ± 1 °C, the pH at 7.0 ± 0.2 with a photoperiod of 14/10 (light/dark). Eggs were obtained by random pairwise mating. Three adult males and four females were placed together in breeding tanks supplied with mesh egg traps to prevent the eggs from being eaten a day prior spawning. Egg harvesting took place the following morning and transferred into a 92 mm plastic Petri dishes containing 40 mL fresh embryo buffer [72]. At 4 hpf any unfertilized eggs that failed cleavage process or eggs showed morphological irregularities during cleavage were discarded. Embryos undergoing normal division process were selected at 6 hpf and utilized for all studies described in this work. Embryos were staged using the pectoral fin, yolk sac, and swim bladder as indicators of developmental stage [73]. Throughout all procedures, embryos and appropriate solutions were kept at 28 ± 1 °C, in the incubator under a light cycle of 14/10 (light/dark).

### 3.3. Embryo Exposure

Healthy embryos were exposed to 2 and 4 μg/mL triadimefon concentrations (Sigma, 45693) of analytical grade dissolved in 0.1% DMSO. Treatment initiated at 6 hpf and continues until the end of each experiment at 120 hpf. Chemical renewal of embryos was repeated every 24 h and carried out in 24-well plates. In each well, 5 embryos were positioned in 2.5 mL of triadimefon solution. Both concentrations of interest were tested in parallel in 6 different wells with up to 3 independent replicates. Concentration selection was based on the initiation of triadimefon phenotypes determined by experiments with reference to a previous publication by Liu et al. [20]. DMSO was used as solvent control at a final concentration of 0.1% *v*/*v*. Phenotypic comparison of 0.1% DMSO solvent and embryo water treated embryos reveals no phenotypic alterations at developmental stages of interest (S7). As a positive control, RA treated embryos were also phenotypically evaluated at known concentrations [34]. For evaluation of embryonic developmental defects the general morphology scoring system was utilized in order to enhance reproducibility and thus improving comparison between experimental groups [74]. Depending on the identified phenotypes morphological assessment of embryonic development at 24, 48, 72, 96 and 120 hpf was carried out enabling detailed monitoring of the developmental time and location where each phenotype arose. During the treatment several lethal or sublethal endpoints including hatching rate, edema, tail detachment, somite formation and heartbeat, were observed and recorded. Zebrafish were anesthetized in ice cold MS222 (200 mg/L) and photographed with a Leica DFC490 digital camera (Leica microsystems LTD, Buffalo Grove, IL, USA) attached to a stereomicroscope (Leica MZ125). Images were displayed using Adobe Photoshop CS6 software (13.0.1.3, Adobe, San Jose, CA, USA).

### 3.4. Effect of Triadimefon on Hatching Rate and Body Length Measurement

To determine whether exposure to triadimefon affected hatching rate, we recorded the percentage of embryos that became free of its chorion in each treatment well at 56, 72 and 77 hpf. In total 90 embryos were observed per treatment group and 0.1% DMSO controls.

Body length of treated embryos and controls was measured to determine triadimefon effect on growth at 120 hpf. Zebrafish were anesthetized in MS222 and digital images were captured using a Leica MZ125 high performance stereomicroscope fitted with a Leica DFC490 digital camera. Fish length was measured from the mouth tip to the tail base and along the body axis utilizing digital images and ImageJ software (version 1.49, National Institutes of Health, Bethesda, MD, USA). Values for body length are presented as mean body length in mm. Sample sizes were *n* = 25 per treatment.

### 3.5. Heart Rate Quantification

Cardiac function was quantified in hearts from each group at 120 hpf and according to Hoage et al., [75]. Briefly, zebrafish embryos were positioned in 3% methylcellulose (Sigma, St. Louis, MO, USA) and hearts were captured in videos for the period of 15 s. Beat rates were later quantified from the digital videos using Leica Application Suite version V4.5.0 software (Leica microsystems LTD). Number of beats was counted and heart rate was calculated by multiplying the number of beats counted by four. The experiment was repeated three times per embryo in a temperature controlled room (27 ± 1 °C). In total 25 embryos per treatment and controls were assessed.

### 3.6. Statistical Analysis

All statistical analyses were undertaken using SPSS 16.0 software. Differences were determined by Kruskal-Wallis test, completed with Kolmogorov-Smirnov test for normality. Significance was determined at *p* = 0.05 (*), *p* = 0.01 (**) and *p* = 0.001 (***).

## 4. Conclusions

The results of the present study demonstrate that zebrafish embryos’ can be used as an alternative animal model for the detection of effects during the embryonic development. The reference substance used to test the suitability of the animal model to respond to developmental toxicants was the fungicide triadimefon, which induced distinct phenotypes particularly at a concentration of 4 μg/mL at 120 hpf. Briefly, significant developmental defects were observed, including delay and inhibition on the hatching rate, reduced body length, spinal curvature, non-depleted yolk sac and uninflated swim bladder. In addition, effects on somite formation and induction of irregularities, disorganized muscle fibers and hypoplastic horizontal myoseptum, hypomorphic midbrain, flattened forebrain, a severe cleft phenotype along the three neural divisions and hypoplastic or even absent mandiblular phenotypes were observed following triadimefon exposure. Interestingly, a massive pericardial edema, abnormal shaped heart, brachycardia and inhibited or even absent embryonic blood circulation were also prominent in exposed zebrafish. The concentration dependence of the produced effects was also observable and significant differences in the incidence and/or severity of the effects were also identified in embryos exposed to different triadimefon concentrations at multiple zebrafish embryonic stages.

Zebrafish larvae developed from embryos treated with triadimefon present severe alterations of the craniofacial structures, derivatives of the first and second branchial arches, skeletal defects, somitic abnormalities, presence of cleft and cardiovascular deficiencies. These observations are very similar to those seen in mammalian models, in vitro studies, *Xenopus* and *ascidian* embryo work as well as those from human observations after exposure to triadimefon and/or other triazole compounds, suggesting that zebrafish can be a reliable model to test azole toxicity to vertebrates. In addition, the similarity of observed phenotypes also suggests common mechanism(s) of action throughout animal models. Therefore, zebrafish has been proved to be an attractive alternative model to study the toxic effects of various compounds since it offers the convenience of working with a vertebrate species and is similar at a physiological level to mammals.

## Figures and Tables

**Table 1:** Summary of phenotypes detected in zebrafish embryos after exposure to 2 and 4 μg/mL triadimefon concentrations.

Phenotype	72 hpf		96 hpf		120 hpf	
	2 µg/mL	4 µg/mL	2 µg/mL	4 µg/mL	2 µg/mL	4 µg/mL
Hatching	--	--	delayed	delayed	delayed	delayed
Length	na	na	na	na	N	shorter
Spine shape	N	N	N	N	N	bent
Yolk sac	N	N	minor enlargement	enlarged	minor enlargement	enlarged/misshaped
Swim bladder	N	N	minor inhibition	minor inhibition	uniflated	uniflated
Somites	N	N	N	N	N	severely affected
Horizontal myoseptum	N	N	N	N	N	reduced
Mandible	N	N	delayed	hypoplastic	severely hypoplastic	severely hypo plastic/absent
Maxilla	N	N	minor delay	minor delay	minor delay	minor delay
Lower pharyngeal arches	N	N	N	possible delay	slightly hypoplastic	severely hypo plastic/absent
Neurocranim	N	N	flattened forebrain	flattened forebrain	mild delay of midbrain/hindbrain	severely reduced size/flattened forebrain hypolastic mid-brain/ no clear forebrain mid-brain boundary mild delay of hindbrain cleft of primary neuronal divisions
Heart shape	N	N	N	linear/delayed	N	massive pericardial edemas severe decrease in size linear shape
Heart rate	na	na	na	na	N	brachycardia
Blood circulation	N	N	N	N	N	severely decrease/absent

na: not assessed, N: normal.

**Table 2:** Overview of phenotypes from zebrafish triadimefon exposure compared to published in vivo, in vitro data and human studies assessing triazole teratogenicity.

Phenotype (Identified/Published)	Zebrafish	Mammalian and Other In Vivo Models	In Vitro Models/Embryo Cultures	Human Studies
Hatching	Inhibited/delayed: could be a result of decrease movement activity due to somitic abnormalities			
Body length	Shorter: may arise from skeletal defects, irregularities in somitic boundaries and disorganized muscle fibers	Malformations at the level of axial skeleton (FON) [13] Fusions, duplications or morphological transformations of mouse axial segments (FON) [12] Reduced humeral length in mouse (FLUC) [63] Axial skeletal defects in mouse (MIC and MET) [64] Reduced body length in ascidian (IM) [17]		Skeletal defects (FLUC) [60] Skeletal manifestations of humeral radial synostosis and femoral bowing (FLUC) [65] Limb defects (IT)
Spine	Bent: phenotype associated with somitic defects that may lead to muscle and skeletal phenotypes	Axial skeletal and limb defects in mouse (FLUS) [66] Axial skeletal defects in mouse (IT) [67] Limb anomalies in rat (KET) [68] Bent spine in ascidian (IM) [17] Axial defects (homeotic respe-cification/lumbar rib) (FON) [44] Short tail in ascidian (IM, FON) [17]		
Yolk sac	Enlarged/misshaped: associated with lipid metabolism defects			
Swim bladder	Uninflated: secondary effects of the curved spine/muscle/skeletal phenotype, a delay in hatching that didn’t allow inflation to occur on time; reduced movement as a result of somitic defects			
Somites	Irregular somitic formation, disorganized muscle fibers, hypolastic horizontal myoseptum	Somite segmentation defects in *X. laevis* (FON, NOL) [16]	Fusion of the I and II branchial arches [7] Romboencephalic cleft, abnormal somites (FON) [49] Abnormal somitic develop-ment (KET and EN) [51]	
Upper branchial arches (facial structures/mandible/maxillae)	Severely hypoplastic or even absent, Minor delay in development	Micrognathia, microtia, short/fused mandible with zygomatic, joined mandible and maxillae, malformed I and II branchial arches (FON) [13] Abnormal shape, fusions, and agenesis of craniofacial structures originated from branchial ectomesenchyme (palatine, basisphenoid, alisphenoid, pterygoid, squamosal, zygomatic, maxilla, mandible, Meckel’s cartilage, tympanic ring, ear ossicles) during mouse development (FON) [12] Absence of mandibular and maxillary cartilages in *X. laevis* (FON) [16] Absence of mandibular cartilage and fusion of mandibular cartilage with adjacent structures in *X. laevis* (NOL) [16] Craniofacial defects in mice (the migration of encephalic neural crest cells) (FON) [44]	Branchial apparatus * reduction/absence, increased cell death of branchial mesenchyme (FLUC and FLU) [50] I branchial arch reduction/I and II branchial arch fusion (FLUC) [57] Reduction of I branchial arch, absence of II branchial arch (FON) [49] Dorso-ventral reduction of I and II branchial arches (NOL) [49] Fusion between I and II branchial arches (FLU, FON and NOL) [69] II branchial arch reduction, I/II branchial arch fusion (KET) [51] I and II branchial arch reduction and fusion (EN) [51] Fusion of the I and II branchial arches (FON) [7] Reduction/ fusion of I and II branchial arches (FON, HEX, FLUS, CYP, MYC, TRI) [70]	
Lower branchial arches	Severely hypoplastic hypoplastic or even absent lower pharyngeal arches		Altered morphogenesis of the branchial apparatus (FLUC) [71] II/III branchial arch fusion (KET) [51]	
Clefts		Cleft palate in rat (FLUC, FON, CYP, KET) [12,47,63,68] Cleft palate in mouse (FLUC, IT, FON) [44,45,63,66]	Cleft forebrain and midbrain (KET) [51]	Cleft palate (FLUC) [60,65] Oral cleft (MIC, TER and KET) [59]
Neurocranium	Reduced size/flat forebrain, hypoplastic midbrain, with no clear forebrain midbrain boundaries	Short anterio-dorsal cranial region, slight mouth protrusion of *X. laevis* (FON, NOL) [16] Alterations of anterior end trunk in ascidian (FON) [17] Abnormal neural crest cell migration from hindbrain to the branchial arches of mice (FLUC) [57]	Reduction of prosencephalon, encephalic schisis (FON) [49] Alterations of hindbrain segmentation (FLU, FON and NOL) [69] Asymmetric forebrain and hindbrain (IMI) [51] Microcephaly, asymmetric forebrain, reduced hindbrain (KET) [51] Reduced, swollen, asymmetric hindbrain (EN) [51]	Craniosynostosis, brachycephaly (FLUC) [60] Craniosynostosis (MIC, TER and KET) [59] Craniosynostosis (FLUC) [65] Craniofacial ossification defects, hypolastic facial bones, small face, brachy-cephaly (FLUC) [60] Microcephalia (IT)
Cardiovascular	Pericardial edema, decrease of heart size, linear heart shape/brachycardia, severely decreased or even absent circulation	Cardiac edema of *X. laevis* (FON, NOL) [16]		Tetralogy of fallot, ventricular septal defects, pulmonary artery hypoplasia (FLUC) [60] Hypoplastic left heart (MIC, TER and KET) [59] Several cardiovascular malformations (FLUC) [65] Hypoplastic left heart syndrome (IT)

* The branchial apparatus is the embryonic structure that gives rise to embryonic craniofacial structures. CYP, cyproconazole; EN, enilconazole; FLUC, fluconazole; FLUS, flusilazole; FLU, flusinazole; HEX, hexaconazole; IM, imazalil; IMI, imidazole; IT, itraconazole; KET, ketoconazole; MET, metronidazole; MIC, miconazole; MYC, myclobutanil; TER, terconazole; FON, triadimefon; NOL, triadimenol; TRI, triticonazole.

## Supplementary Materials

Supplementary materials can be found at www.mdpi.com/1422-0067/18/4/817/s1.

## Abbreviations

hpf	Hours post-fertilization
RA	Retinoic acid
WEC	Whole embryo culture
NCC	Neural crest cell
